# The genome sequence of the Western Capercaillie *Tetrao urogallus *Linnaeus, 1758

**DOI:** 10.12688/wellcomeopenres.21261.1

**Published:** 2024-04-15

**Authors:** Alex Ball, Carolyn Robertson, Molly Doubleday, Wellcome Sanger Institute Tree of Life Management, Samples and Laboratory team, Wellcome Sanger Institute Scientific Operations: Sequencing Operations, Wellcome Sanger Institute Tree of Life Core Informatics team, Tree of Life Core Informatics collective, Darwin Tree of Life Consortium

**Affiliations:** 1 RZSS WildGenes, Royal Zoological Society of Scotland, Edinburgh, Scotland, UK; 2 Cairngorms National Park Authority, Grantown on Spey, Scotland, UK; 3 Royal Society for the Protection of Birds (RSPB) Scotland, Edinburgh, Scotland, UK

**Keywords:** Tetrao urogallus, Western Capercaillie, genome sequence, chromosomal, Galliformes

## Abstract

We present a genome assembly from an individual male *Tetrao urogallus* (the Western Capercaillie; Chordata; Aves; Galliformes; Phasianidae). The genome sequence is 1,013.2 megabases in length. Most of the assembly is scaffolded into 39 chromosomal pseudomolecules, including the Z sex chromosome. The mitochondrial genome has also been assembled and is 16.68 kilobases in length.

## Species taxonomy

Eukaryota; Opisthokonta; Metazoa; Eumetazoa; Bilateria; Deuterostomia; Chordata; Craniata; Vertebrata; Gnathostomata; Teleostomi; Euteleostomi; Sarcopterygii; Dipnotetrapodomorpha; Tetrapoda; Amniota; Sauropsida; Sauria; Archelosauria; Archosauria; Dinosauria; Saurischia; Theropoda; Coelurosauria; Aves; Neognathae; Galloanserae; Galliformes; Phasianidae; Tetraoninae; *Tetrao*; *Tetrao urogallus* Linnaeus, 1758 (NCBI:txid100830).

## Background

**Figure 1.**
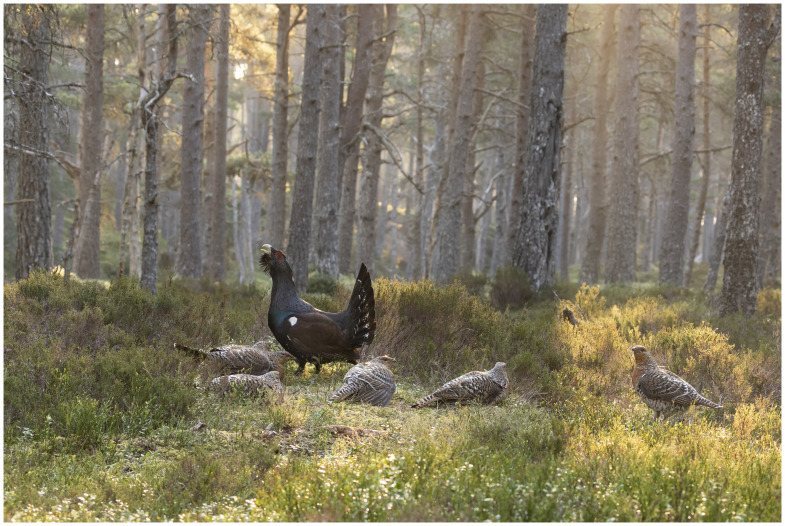
Scottish lek site in Caledonian pine forest; displaying male with 6 females. Credit Mark Hamblin.

The Western Capercaillie ( *Tetrao urogallus*) ( Figure 1) is the world’s largest grouse species. It is found in mixed coniferous forests across Eurasia, from northern Spain through to Russia. While populations in the northern parts of its range, including Scandinavia and Russia are large, there have been dramatic declines in central and western Europe likely due to habitat fragmentation and hunting. In the UK, the capercaillie was previously driven to extinction in the 18th century, and the present-day population is the result of a successful reintroduction in the UK, which occurred in Scotland during the 1830s ( Stevenson, 2007). However, after reaching an estimated population size of 20,000 birds in the 1970s, the capercaillie is once again facing extinction in the UK ( Moss *et al.*, 2000; Wilkinson *et al.*, 2024). Huge declines have occurred in the last few decades, with the most recent national survey estimating that only 532 birds remain ( Wilkinson *et al.*, 2024). Since the first UK listings of ‘Birds of conservation concern’ in 1996, the capercaillie has been included as a red-list priority species.

The capercaillie’s unusual mating system consists of spring leks, in which males congregate to display via dances, clicking and popping sounds in open forest areas. These sounds are thought to have led to its name in Gaelic, *capall coille*, meaning ‘horse of the woods’. These displays, its historic status as a game bird and its association with the last remaining wild Caledonian pine forests has led to its iconic status in Scotland.

Capercaillie require extensive areas of Scots pine ( *Pinus sylvestris*) dominated woodland, which in the UK, is only available in Scotland. This specific habitat need make **s** capercaillie particularly vulnerable to habitat fragmentation and unfavourable forest management. Limited and fragmented habitat is a recognised cause of population decline and can lead capercaillie populations to become isolated. Less habitat means capercaillie may also be more prone to the impacts of predation and human disturbance. The Review of Capercaillie Conservation and Management commissioned by NatureScot in 2021 included consideration of several fundamental issues facing the species, including predation and human disturbance ( NatureScot, 2022). Mortality associated with deer fence collisions and reduced breeding success associated with high April temperatures and high June rainfall were also cited as fundamental issues.

With the UK capercaillie population now at a critically low level, the Cairngorms National Park Authority and NatureScot are tasked with bringing together stakeholders from across the spectrum to explore a range of options to help the species. This includes coordinating activities from fence marking and removal, to working with access takers, expanding pinewood habitat at landscape scale and exploring the feasibility of reinforcement.

An adaptive, evidence-led approach to improve management for this species is the ultimate aim, and a key part of this approach has been the generation of genomic tools to improve management decisions. The RZSS WildGenes team at the Royal Zoological Society of Scotland has generated genetic data from tissue and blood samples from across Europe to create a panel of target enrichment probes that can be applied to degraded but non-invasively collected feather and faecal samples. The collection of these sample types minimises disturbance to the remaining birds and will provide increased insights to the currently used field approaches. The new panel of genomic markers are being used to investigate geographic origin of the capercaillie in Scotland, population structure, genetic diversity and individual identification. The use of the enrichment probes, however, relies on mapping of sequences to a reference genome, until now the Greater prairie chicken genome has been used with limited success. The generation of a capercaillie genome will now allow researchers to identify a greater number of variable markers within this species, increasing our ability to monitor the population in future. By identifying unique individual genetic signatures in the samples, the aim is to improve the accuracy of current population estimates, not only in Scotland, but across Europe.

## Genome sequence report

The genome was sequenced from a male *Tetrao urogallus* found deceased in Cairngorms, Scotland, UK. A total of 23-fold coverage in Pacific Biosciences single-molecule HiFi long reads was generated. Primary assembly contigs were scaffolded with chromosome conformation Hi-C data. Manual assembly curation corrected 66 missing joins or mis-joins and removed 2 haplotypic duplications, reducing the scaffold number by 7.29%.

**Figure 2.**
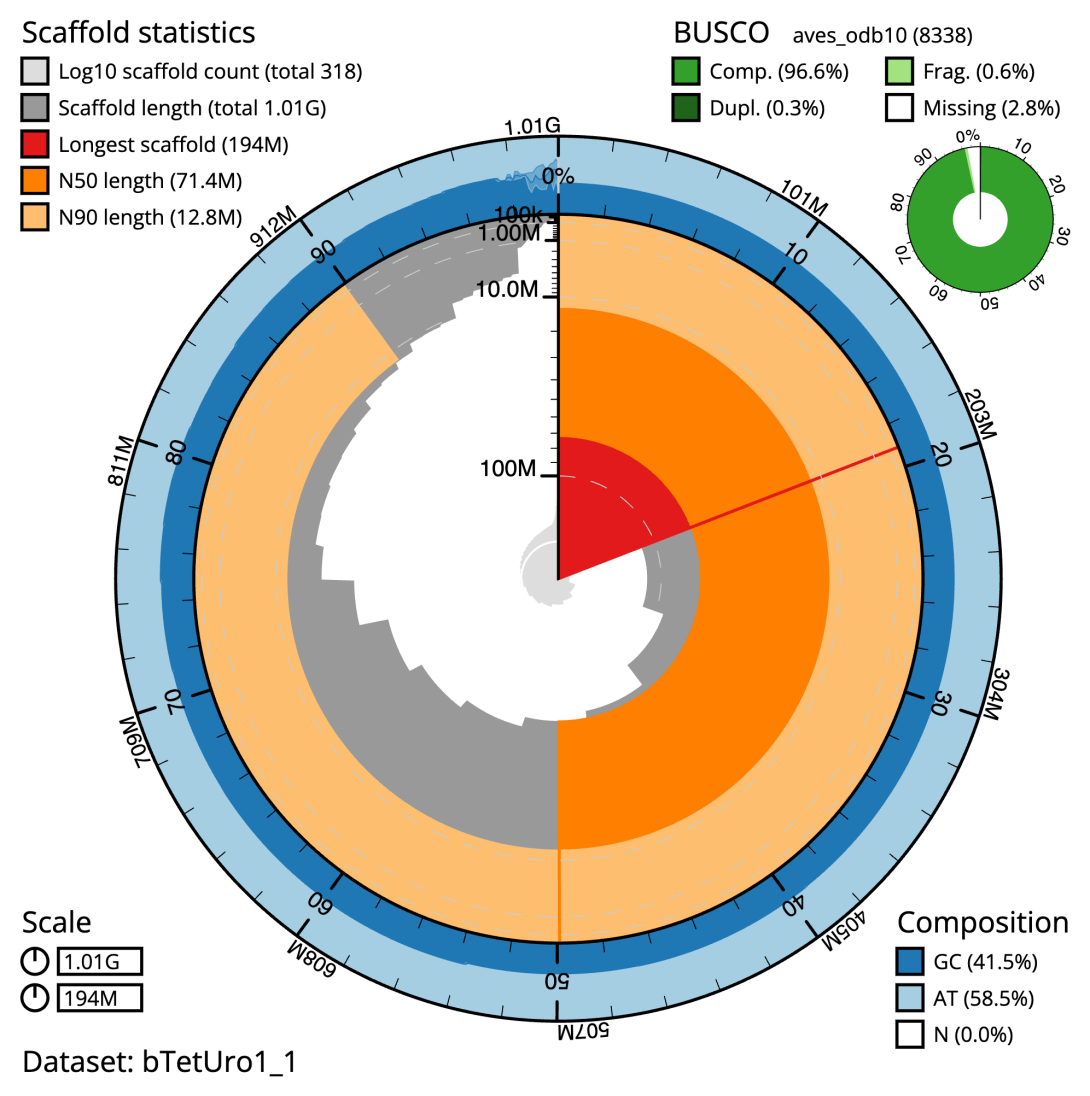
Genome assembly of *Tetrao urogallus*, bTetUro1.1: metrics. The BlobToolKit snail plot shows N50 metrics and BUSCO gene completeness. The main plot is divided into 1,000 size-ordered bins around the circumference with each bin representing 0.1% of the 1,013,184,029 bp assembly. The distribution of scaffold lengths is shown in dark grey with the plot radius scaled to the longest scaffold present in the assembly (193,917,226 bp, shown in red). Orange and pale-orange arcs show the N50 and N90 scaffold lengths (71,401,156 and 12,783,881 bp), respectively. The pale grey spiral shows the cumulative scaffold count on a log scale with white scale lines showing successive orders of magnitude. The blue and pale-blue area around the outside of the plot shows the distribution of GC, AT and N percentages in the same bins as the inner plot. A summary of complete, fragmented, duplicated and missing BUSCO genes in the aves_odb10 set is shown in the top right. An interactive version of this figure is available at https://blobtoolkit.genomehubs.org/view/bTetUro1_1/dataset/bTetUro1_1/snail.

**Figure 3.**
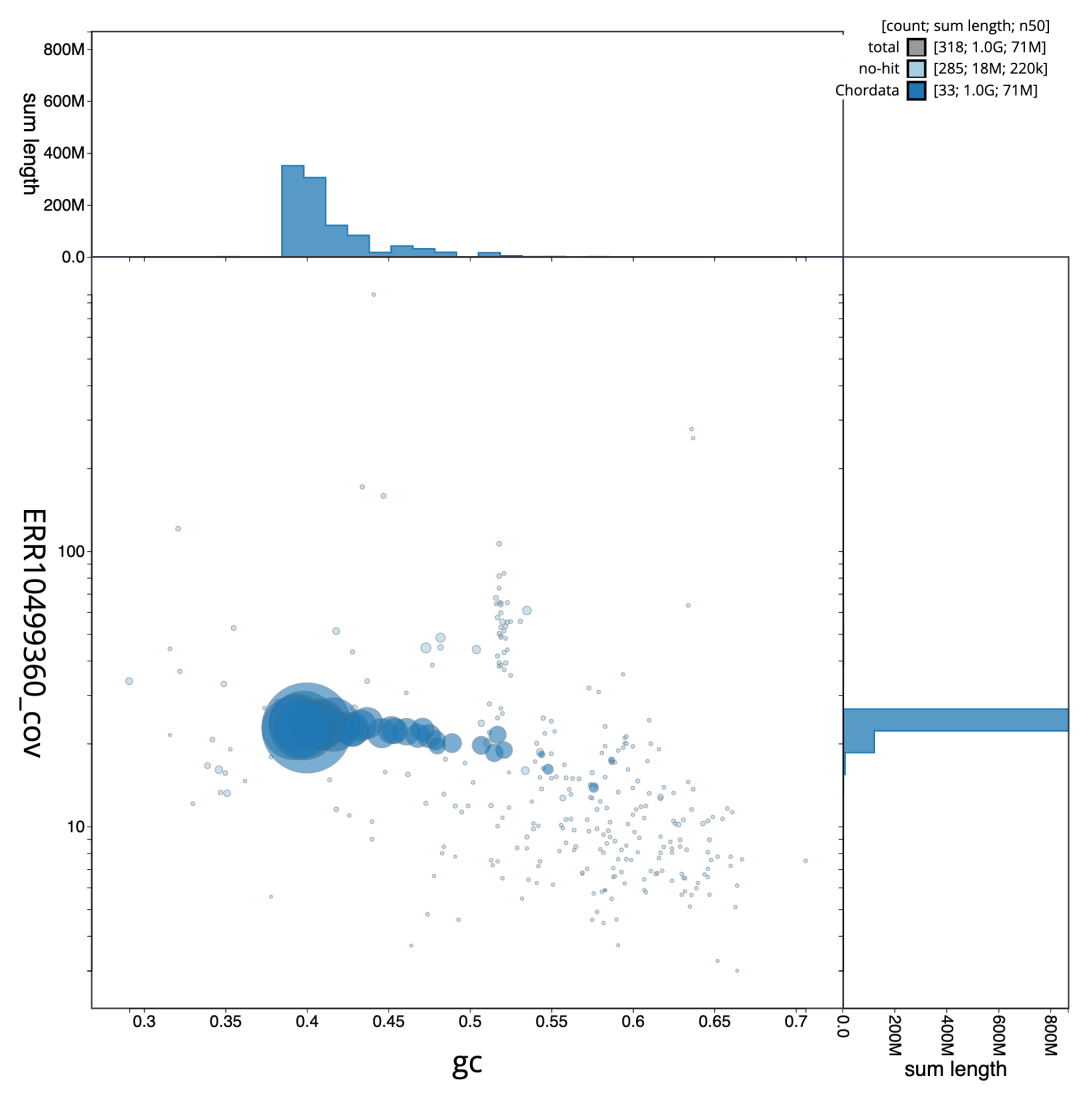
Genome assembly of *Tetrao urogallus*, bTetUro1.1: BlobToolKit GC-coverage plot. Sequences are coloured by phylum. Circles are sized in proportion to sequence length. Histograms show the distribution of sequence length sum along each axis. An interactive version of this figure is available at https://blobtoolkit.genomehubs.org/view/bTetUro1_1/dataset/bTetUro1_1/blob.

**Figure 4.**
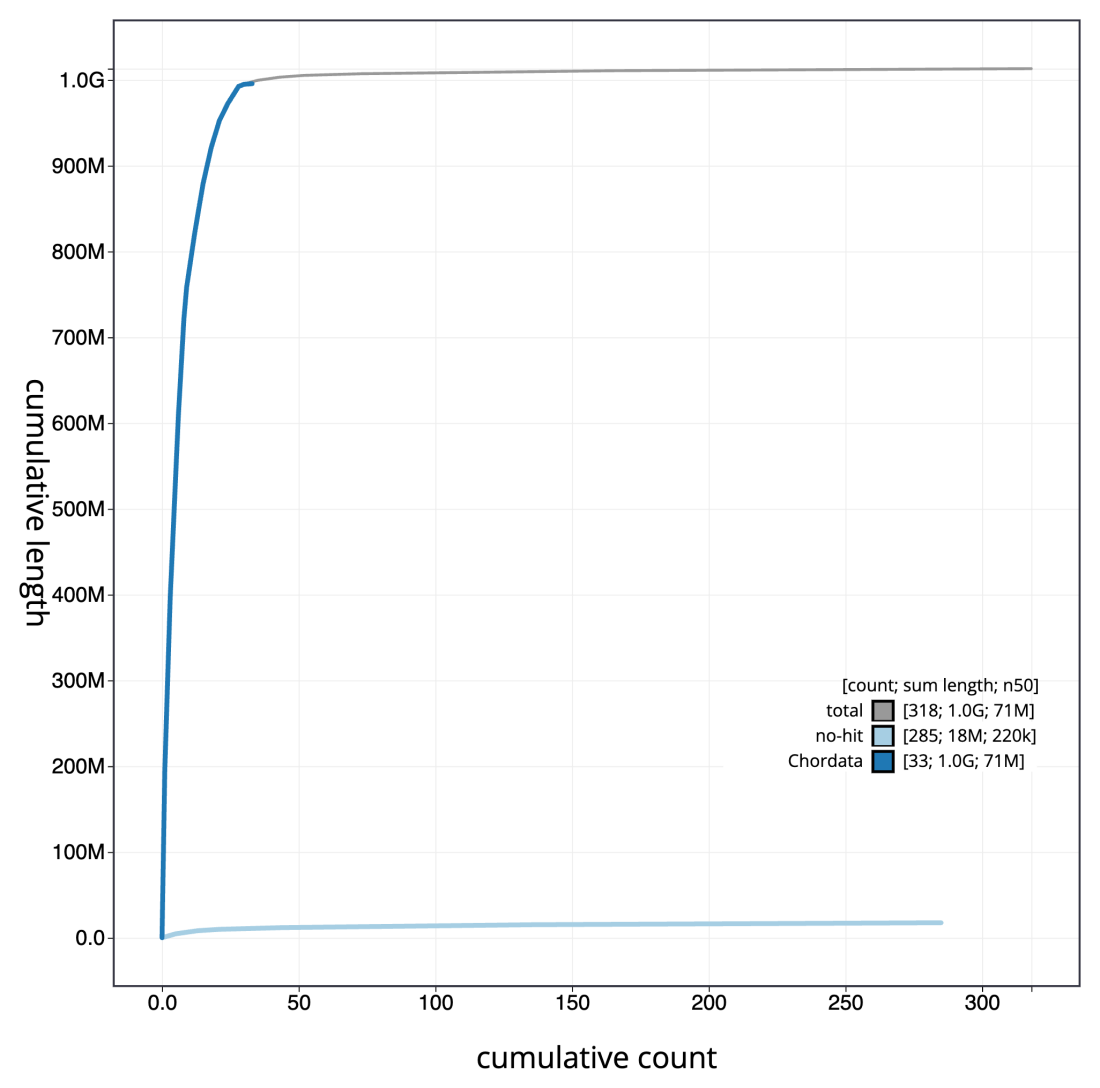
Genome assembly of *Tetrao urogallus*, bTetUro1.1: BlobToolKit cumulative sequence plot. The grey line shows cumulative length for all sequences. Coloured lines show cumulative lengths of sequences assigned to each phylum using the buscogenes taxrule. An interactive version of this figure is available at https://blobtoolkit.genomehubs.org/view/bTetUro1_1/dataset/bTetUro1_1/cumulative.

**Figure 5.**
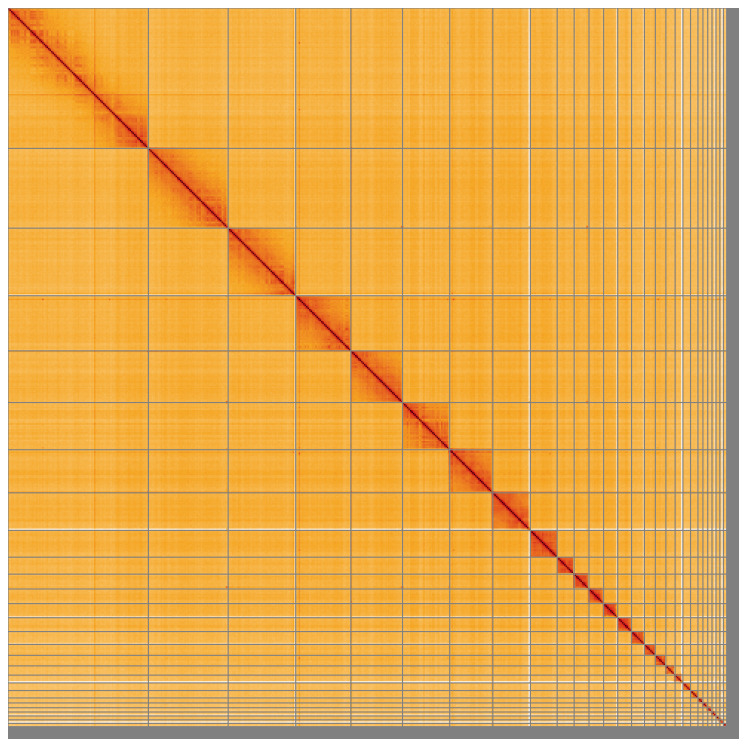
Genome assembly of *Tetrao urogallus*, bTetUro1.1: Hi-C contact map of the bTetUro1.1 assembly, visualised using HiGlass. Chromosomes are shown in order of size from left to right and top to bottom. An interactive version of this figure may be viewed at https://genome-note-higlass.tol.sanger.ac.uk/l/?d=JQ17gbhIQJWxkHqBdByFYQ.

The final assembly has a total length of 1,013.2 Mb in 317 sequence scaffolds with a scaffold N50 of 71.4 Mb ( Table 1). The snail plot in Figure 2 provides a summary of the assembly statistics, while the distribution of assembly scaffolds on GC proportion and coverage is shown in Figure 3. The cumulative assembly plot in Figure 4 shows curves for subsets of scaffolds assigned to different phyla. Most (99%) of the assembly sequence was assigned to 39 chromosomal-level scaffolds, representing 38 autosomes and the Z sex chromosome. Chromosome-scale scaffolds confirmed by the Hi-C data are named in order of size ( Figure 5; Table 2). The Z sex chromosome was identified by alignment to *Gallus gallus* (GCA_016699485.1). While not fully phased, the assembly deposited is of one haplotype. Contigs corresponding to the second haplotype have also been deposited. The mitochondrial genome was also assembled and can be found as a contig within the multifasta file of the genome submission.

**Table 1.** Genome data for *Tetrao urogallus*, bTetUro1.1.

Project accession data		
Assembly identifier	bTetUro1.1	
Species	*Tetrao urogallus*	
Specimen	bTetUro1	
NCBI taxonomy ID	100830	
BioProject	PRJEB57676	
BioSample ID	SAMEA9654429	
Isolate information	bTetUro1, muscle (DNA and Hi-C sequencing)	
Assembly metrics *		*Benchmark*
Consensus quality (QV)	59.5	*≥ 50*
*k*-mer completeness	100.0%	*≥ 95%*
BUSCO **	C:96.6%[S:96.3%,D:0.3%],F:0.6%,M:2.8%,n:8,338	*C ≥ 95%*
Percentage of assembly mapped to chromosomes	99%	*≥ 95%*
Sex chromosomes	Z	*localised homologous pairs*
Organelles	Mitochondrial genome: 16.68 kb	*complete single alleles*
Raw data accessions		
PacificBiosciences SEQUEL II	ERR10499360	
Hi-C Illumina	ERR10501026	
Genome assembly		
Assembly accession	GCA_951394365.1	
*Accession of alternate haplotype*	GCA_951394355.1	
Span (Mb)	1,013.2	
Number of contigs	1,291	
Contig N50 length (Mb)	1.9	
Number of scaffolds	317	
Scaffold N50 length (Mb)	71.4	
Longest scaffold (Mb)	193.92	

* Assembly metric benchmarks are adapted from column VGP-2020 of “Table 1: Proposed standards and metrics for defining genome assembly quality” from Rhie *et al.* (2021). ** BUSCO scores based on the aves_odb10 BUSCO set using version 5.3.2. C = complete [S = single copy, D = duplicated], F = fragmented, M = missing, n = number of orthologues in comparison. A full set of BUSCO scores is available at https://blobtoolkit.genomehubs.org/view/bTetUro1_1/dataset/bTetUro1_1/busco.

**Table 2.** Chromosomal pseudomolecules in the genome assembly of *Tetrao urogallus*, bTetUro1.

INSDC accession	Chromosome	Length (Mb)	GC%
OX596294.1	1	193.92	40.0
OX596295.1	2	110.12	40.0
OX596296.1	3	93.28	39.0
OX596298.1	4	71.4	39.5
OX596299.1	5	65.17	41.5
OX596300.1	6	59.51	41.0
OX596301.1	7	51.87	40.5
OX596302.1	8	37.0	41.0
OX596303.1	9	23.44	43.0
OX596304.1	10	20.48	43.5
OX596305.1	11	20.2	43.5
OX596306.1	12	19.44	42.5
OX596307.1	13	19.16	43.0
OX596308.1	14	17.78	44.5
OX596309.1	15	15.03	45.0
OX596310.1	16	14.53	46.0
OX596311.1	17	12.78	45.5
OX596312.1	18	10.72	47.0
OX596313.1	19	10.6	47.5
OX596314.1	20	10.18	47.0
OX596315.1	21	6.89	48.0
OX596316.1	22	6.65	49.0
OX596317.1	23	5.89	50.5
OX596318.1	24	5.46	51.5
OX596319.1	25	5.33	51.5
OX596320.1	26	4.9	52.0
OX596321.1	27	4.55	48.0
OX596322.1	28	1.38	55.0
OX596323.1	29	0.72	50.5
OX596324.1	30	1.31	47.5
OX596325.1	31	1.15	57.5
OX596326.1	32	1.01	48.0
OX596327.1	33	0.84	53.5
OX596328.1	34	0.76	51.0
OX596329.1	35	0.62	53.5
OX596330.1	36	0.47	54.5
OX596331.1	37	0.23	55.5
OX596332.1	38	0.11	63.0
OX596297.1	Z	76.13	40.0
OX596333.1	MT	0.02	44.0

The estimated Quality Value (QV) of the final assembly is 59.5 with *k*-mer completeness of 100.0%, and the assembly has a BUSCO v5.3.2 completeness of 96.6% (single = 96.3%, duplicated = 0.3%), using the aves_odb10 reference set ( *n* = 8,338).

Metadata for specimens, barcode results, spectra estimates, sequencing runs, contaminants and pre-curation assembly statistics are given at https://tolqc.cog.sanger.ac.uk/darwin/birds/Tetrao_urogallus/.

## Methods

### Sample acquisition

A male capercaillie carcass (specimen ID SAN0001380, ToLID bTetUro1) was found in Anagach Wood, Strathspey, Scotland on 2020-06-01. The carcass weighed 3.72 kg and was stored frozen at –20°C until a post-mortem was conducted on 2020-08-04. The post-mortem was unable to identify a cause of death, however a skeletal muscle tissue sample was taken and placed in 90% ethanol. This sample was stored at –20°C until transfer to Darwin Tree of Life. The carcass was collected and identified by Molly Doubleday, RSPB Capercaillie Advisory Officer.

### Sample preparation and nucleic acid extraction

The workflow for high molecular weight (HMW) DNA extraction at the Wellcome Sanger Institute (WSI) includes a sequence of core procedures: sample preparation; sample homogenisation, DNA extraction, fragmentation, and clean-up. In sample preparation, the bTetUro1 sample was weighed and dissected on dry ice ( Jay *et al.*, 2023). For sample homogenisation, muscle tissue was cryogenically disrupted using the Covaris cryoPREP ^®^ Automated Dry Pulverizer ( Narváez-Gómez *et al.*, 2023).

HMW DNA was extracted using the Automated MagAttract v2 protocol ( Oatley *et al.*, 2023a). DNA was sheared into an average fragment size of 12–20 kb in a Megaruptor 3 system ( Bates *et al.*, 2023). Sheared DNA was purified by solid-phase reversible immobilisation ( Oatley *et al.*, 2023b): in brief, the method employs a 1.8X ratio of AMPure PB beads to sample to eliminate shorter fragments and concentrate the DNA. The concentration of the sheared and purified DNA was assessed using a Nanodrop spectrophotometer and Qubit Fluorometer and Qubit dsDNA High Sensitivity Assay kit. Fragment size distribution was evaluated by running the sample on the FemtoPulse system.

Protocols developed by the WSI Tree of Life laboratory are publicly available on protocols.io ( Denton *et al.*, 2023).

### Sequencing

Pacific Biosciences HiFi circular consensus DNA sequencing libraries were constructed according to the manufacturers’ instructions. DNA sequencing was performed by the Scientific Operations core at the WSI on a Pacific Biosciences SEQUEL II instrument. Hi-C data were also generated from muscle tissue of bTetUro1 using the Arima2 kit and sequenced on the Illumina NovaSeq 6000 instrument.

### Genome assembly, curation and evaluation

Assembly was carried out with Hifiasm ( Cheng *et al.*, 2021) and haplotypic duplication was identified and removed with purge_dups ( Guan *et al.*, 2020). The assembly was then scaffolded with Hi-C data ( Rao *et al.*, 2014) using YaHS ( Zhou *et al.*, 2023). The assembly was checked for contamination and corrected as described previously ( Howe *et al.*, 2021). Manual curation was performed using HiGlass ( Kerpedjiev *et al.*, 2018) and PretextView ( Harry, 2022). The mitochondrial genome was assembled using MitoHiFi ( Uliano-Silva *et al.*, 2023), which runs MitoFinder ( Allio *et al.*, 2020) or MITOS ( Bernt *et al.*, 2013) and uses these annotations to select the final mitochondrial contig and to ensure the general quality of the sequence.

A Hi-C map for the final assembly was produced using bwa-mem2 ( Vasimuddin *et al.*, 2019) in the Cooler file format ( Abdennur & Mirny, 2020). To assess the assembly metrics, the *k*-mer completeness and QV consensus quality values were calculated in Merqury ( Rhie *et al.*, 2020). This work was done using Nextflow ( Di Tommaso *et al.*, 2017) DSL2 pipelines “sanger-tol/readmapping” ( Surana *et al.*, 2023a) and “sanger-tol/genomenote” ( Surana *et al.*, 2023b). The genome was analysed within the BlobToolKit environment ( Challis *et al.*, 2020) and BUSCO scores ( Manni *et al.*, 2021; Simão *et al.*, 2015) were calculated.

Table 3 contains a list of relevant software tool versions and sources.

**Table 3.** Software tools: versions and sources.

Software tool	Version	Source
BlobToolKit	4.1.7	https://github.com/blobtoolkit/blobtoolkit
BUSCO	5.3.2	https://gitlab.com/ezlab/busco
Hifiasm	0.16.1-r375	https://github.com/chhylp123/hifiasm
HiGlass	1.11.6	https://github.com/higlass/higlass
Merqury	MerquryFK	https://github.com/thegenemyers/MERQURY.FK
MitoHiFi	2	https://github.com/marcelauliano/MitoHiFi
PretextView	0.2	https://github.com/wtsi-hpag/PretextView
purge_dups	1.2.3	https://github.com/dfguan/purge_dups
sanger-tol/ genomenote	v1.0	https://github.com/sanger-tol/genomenote
sanger-tol/ readmapping	1.1.0	https://github.com/sanger-tol/readmapping/tree/1.1.0
YaHS	1.2a	https://github.com/c-zhou/yahs

### Wellcome Sanger Institute – Legal and Governance

The materials that have contributed to this genome note have been supplied by a Darwin Tree of Life Partner. The submission of materials by a Darwin Tree of Life Partner is subject to the **‘Darwin Tree of Life Project Sampling Code of Practice’**, which can be found in full on the Darwin Tree of Life website here. By agreeing with and signing up to the Sampling Code of Practice, the Darwin Tree of Life Partner agrees they will meet the legal and ethical requirements and standards set out within this document in respect of all samples acquired for, and supplied to, the Darwin Tree of Life Project.

Further, the Wellcome Sanger Institute employs a process whereby due diligence is carried out proportionate to the nature of the materials themselves, and the circumstances under which they have been/are to be collected and provided for use. The purpose of this is to address and mitigate any potential legal and/or ethical implications of receipt and use of the materials as part of the research project, and to ensure that in doing so we align with best practice wherever possible. The overarching areas of consideration are:

• Ethical review of provenance and sourcing of the material

• Legality of collection, transfer and use (national and international) 

Each transfer of samples is further undertaken according to a Research Collaboration Agreement or Material Transfer Agreement entered into by the Darwin Tree of Life Partner, Genome Research Limited (operating as the Wellcome Sanger Institute), and in some circumstances other Darwin Tree of Life collaborators.

## Data Availability

European Nucleotide Archive: *Tetrao urogallus* (western capercaillie). Accession number PRJEB57676; https://identifiers.org/ena.embl/PRJEB57676 ( Wellcome Sanger Institute, 2023). The genome sequence is released openly for reuse. The *Tetrao urogallus* genome sequencing initiative is part of the Darwin Tree of Life (DToL) project and the European Reference Genome Atlas Pilot Project (ERGA-Pilot) (Bioproject ID PRJEB47820). The assembly is provided by the Wellcome Sanger Institute Tree of Life Programme in collaboration with Peter Klinga (Technical University in Zvolen in Slovakia, species ambassador ERGA) and the European Reference Genome Atlas Pilot Project team. All raw sequence data and the assembly have been deposited in INSDC databases. The genome will be annotated using available RNA-Seq data and presented through the Ensembl pipeline at the European Bioinformatics Institute. Raw data and assembly accession identifiers are reported in Table 1.

## Author information

Members of the Darwin Tree of Life Barcoding collective are listed here: https://doi.org/10.5281/zenodo.4893703.

Members of the Wellcome Sanger Institute Tree of Life Management, Samples and Laboratory team are listed here: https://doi.org/10.5281/zenodo.10066175.

Members of Wellcome Sanger Institute Scientific Operations: Sequencing Operations are listed here: https://doi.org/10.5281/zenodo.10043364.

Members of the Wellcome Sanger Institute Tree of Life Core Informatics team are listed here: https://doi.org/10.5281/zenodo.10066637.

Members of the Tree of Life Core Informatics collective are listed here: https://doi.org/10.5281/zenodo.5013541.

Members of the Darwin Tree of Life Consortium are listed here: https://doi.org/10.5281/zenodo.4783558.
