# Supplementary material for: The genome sequence of the Western Capercaillie Tetrao urogallus Linnaeus, 1758
Source: Wellcome Open Res. 2024 Apr 15;9:198. [Version 1] doi: 10.12688/wellcomeopenres.21261.1 (PMC11066522; doi:10.12688/wellcomeopenres.21261.1)
Supplement: Peer review history

# Reviewer report: Reviewer response for version 1

**DOI:** 10.21956/wellcomeopenres.23514.r80820

**Published:** 2024-05-02

**Authors:** Ruiqi Li

**Affiliations:** 1 University of Colorado Boulder, Boulder, Colorado, USA

The genome note is well-written, offering comprehensive insights into the species’ ecology, biology, and conservation status. The genome quality is excellent. I only have a few minor suggestions:

1. It would be informative to mention the reasons for the species’ recent decline in the UK, providing readers with context regarding current threats.
2. When referencing "aves_odb10", please include the full designation as “avian database (aves_odb10)” to ensure clarity for readers who may not be familiar with BUSCO databases.
3. Has karyotyping been performed to verify the chromosome count?
4. The use of the Ensembl pipeline for annotation is only briefly mentioned in the data availability section. It would be beneficial to discuss this more prominently in the methods and results sections, including details such as the number of genes identified.

Are sufficient details of methods and materials provided to allow replication by others?

Yes

Is the rationale for creating the dataset(s) clearly described?

Yes

Are the datasets clearly presented in a useable and accessible format?

Yes

Are the protocols appropriate and is the work technically sound?

Yes

Reviewer Expertise:

Genomics

I confirm that I have read this submission and believe that I have an appropriate level of expertise to confirm that it is of an acceptable scientific standard.

# Reviewer report: Reviewer response for version 1

**DOI:** 10.21956/wellcomeopenres.23514.r80822

**Published:** 2024-04-30

**Authors:** Tyler Alioto

**Affiliations:** 1 Fundacion Centro Nacional de Analisi Genomico (Ringgold ID: 478092), Barcelona, Catalonia, Spain

The genome note submitted by Ball et al. reports the genome sequence of the Western Capercaillie, *Tetrao urogallus*. Although Northern populations are large, this grouse species is endangered in the UK, with less than a thousand individuals estimated remaining. The assembly is chromosome-scale and of high quality, meeting the minimum standards recommended by the Earth Biogenome Project. All protocols are appropriate and well-documented. All data conforms with FAIR principles, with read data and assemblies being available in the ENA. Blobtoolkit figures are interactive. Additional QC data are provided at https://tolqc.cog.sanger.ac.uk/, supplementing the figures published in the data note.

Are sufficient details of methods and materials provided to allow replication by others?

Yes

Is the rationale for creating the dataset(s) clearly described?

Yes

Are the datasets clearly presented in a useable and accessible format?

Yes

Are the protocols appropriate and is the work technically sound?

Yes

Reviewer Expertise:

genomics, genome assembly, gene prediction, annotation

I confirm that I have read this submission and believe that I have an appropriate level of expertise to confirm that it is of an acceptable scientific standard.
